# Correlation Study of ^18^F-Fluorodeoxyglucose Positron Emission Tomography/Computed Tomography in Pathological Subtypes of Invasive Lung Adenocarcinoma and Prognosis

**DOI:** 10.3389/fonc.2019.00908

**Published:** 2019-09-18

**Authors:** Bin Yang, Hengshan Ji, Yingqian Ge, Sui Chen, Hong Zhu, Guangming Lu

**Affiliations:** ^1^Department of Medical Imaging, Jinling Hospital, Medical School of Nanjing University, Nanjing, China; ^2^Department of Nuclear Medicine, Jinling Hospital, Medical School of Nanjing University, Nanjing, China; ^3^Siemens Healthineers Ltd., Shanghai, China

**Keywords:** lung adenocarcinoma, pathological subtype, FDG PET/CT, metabolic parameters, prognosis

## Abstract

**Purpose:** To investigate the correlation between ^18^F-fluorodeoxyglucose positron emission tomography/computed tomography (^18^F-FDG PET/CT) metabolic parameters and clinicopathological factors in pathological subtypes of invasive lung adenocarcinoma and prognosis.

**Patients and Methods:** Metabolic parameters and clinicopathological factors from 176 consecutive patients with invasive lung adenocarcinoma between August 2008 and August 2016 who underwent ^18^F-FDG PET/CT examination were retrospectively analyzed. Invasive lung adenocarcinoma was divided into five pathological subtypes:lepidic predominant adenocarcinoma (LPA), acinar predominant adenocarcinoma (APA), papillary predominant adenocarcinoma (PPA), solid predominant adenocarcinoma (SPA), and micropapillary predominant adenocarcinoma (MPA). The differences in metabolic parameters [maximal standard uptake value (SUVmax), mean standard uptake value (SUVmean), total lesion glycolysis (TLG), and metabolic tumor volume (MTV)] and tumor diameter for different pathological subtypes were analyzed. Patients were divided into two groups according to their prognosis: good prognosis group (LPA, APA, PPA) and poor prognosis group (SPA, MPA). Logistic regression was used to filter predictors and construct a predictive model, and areas under the receiver operating curve (AUC) were calculated. Cox regression analysis was performed on prognostic factors.

**Results:** 82 (46.6%) females and 94 (53.4%) males of patients with invasive lung adenocarcinoma were enrolled in this study. Metabolic parameters and tumor diameter of different pathological subtype had statistically significant (*P* < 0.05). The predictive model constructed using independent predictors (Distant metastasis, Ki-67, and SUVmax) had good classification performance for both groups. The AUC for SUVmax was 0.694 and combined with clinicopathological factors were 0.745. Cox regression analysis revealed that Stage, TTF-1, MTV, and pathological subtype were independent risk factors for patient prognosis. The hazard ratio (HR) of the poor prognosis group was 1.948 (95% CI 1.042–3.641) times the good prognosis group. The mean survival times of good and poor prognosis group were 50.2621 (95% CI 47.818–52.706) and 35.8214 (95% CI 27.483–44.159) months, respectively, while the median survival time was 47.00 (95% CI 45.000–50.000) and 31.50 (95% CI 23.000–49.000) months, respectively.

**Conclusion:** PET/CT metabolic parameters combined with clinicopathological factors had good classification performance for the different pathological subtypes, which may provide a reference for treatment strategies and prognosis evaluation of patients.

## Introduction

Adenocarcinoma is the most common type of lung cancer, with a high incidence among women and is the most common type of lung cancer in non-smokers (1–4). The incidence and mortality of lung adenocarcinoma in China are on the rise, and rank first among all malignant tumors and are considered to be the most threatening to human health (5). Moreover, lung cancer patients lack obvious clinical signs and symptoms in the early stages. Most lung cancer patients are diagnosed at an advanced stage, resulting in a low 5-year survival rate (6). The histological classification used for lung cancer in the past cannot meet the needs of clinical treatment or predictive prognosis, nor can it reflect progress in imaging, pathology and tumor molecular biology. Furthermore, because most lung adenocarcinomas are the mixed subtype, it is now believed that mixed subtypes should not be classified as an independent histology but be classified according to the major histological subtypes (7–9). Therefore, the International Association for the Study of Lung Cancer (IASLC), American Thoracic Society (ATS), and European Respiratory Society (ERS) first proposed a new international classification standard for lung adenocarcinoma in 2011 (10–12), which divided adenocarcinoma into four types: pre-invasive lesions, microinvasive adenocarcinoma (MIA), invasive adenocarcinoma and invasive adenocarcinoma variants. Among these, pre-invasive lesions are divided into atypical adenomatous hyperplasia (AAH) and adenocarcinoma *in situ* (AIS). Invasive adenocarcinoma (IAC) was divided into lepidic predominant adenocarcinoma (LPA), papillary predominant adenocarcinoma (PPA), and acinar predominant adenocarcinoma (APA), in addition to solid predominant adenocarcinoma (SPA), and micropapillary predominant adenocarcinoma (MPA).

More recent studies have shown that the histological subtype of lung adenocarcinoma in the new classification was closely related to patient prognosis. Some studies have reported that in the new classification method, different lung adenocarcinoma subtypes have different 3- and 5-year disease-free survival rates (13–15). Gu et al. (16) and Yoshizawa et al. (17) reported that the 5-year disease-free survival rate of AIS and MIA can reach 100%. Therefore, some investigators have considered AIS and MIA to be low-grade cancers, while IAC is a medium-grade or advanced cancer (18). The prognosis of invasive lung cancer with different pathological subtypes is different. It is generally considered that the prognosis for LPA, PPA, and APA is better, while the prognosis for SPA and MPA is poor. Therefore, the classification of different pathological subtypes by non-invasive methods prior to surgery is critical to the patient's treatment and prognosis.

However, most current studies have mainly addressed the identification of pre-invasive and invasive lesions. Computed tomography (CT) examination has been used to identify preinvasive and invasive lesions of the nodules by combining morphological features of the nodules with quantitative CT parameters (such as mean CT value and CT number histogram) (19–21). Given the advances in radiomics, some investigators are now using CT texture analysis and radiomics to identify pre-invasive and invasive lesions (22–24). However, there have been few studies addressing the identification of different pathological subtypes of invasive lung adenocarcinoma. Furthermore, using ^18^F-fluorodeoxyglucose positron emission tomography/computed tomography (^18^F-FDG PET/CT) metabolic parameters and clinicopathological factors to identify different pathological subtypes and prognosis has been less reported.

Therefore, the purpose of this study was to use PET/CT metabolic parameters and clinicopathological factors to study the correlation between the pathological subtypes of invasive lung adenocarcinoma and prognosis, which may provide a reference for treatment strategies and prognosis evaluation.

## Patients And Methods

### Patients

The institutional review board of Jinling Hospital, Medical School of Nanjing University approved this retrospective study and waived the requirement for informed consent due to the nature of the study. Clinicopathological factors from 176 consecutive patients with invasive lung adenocarcinoma collected between August 2008 and August 2016 were retrospectively analyzed. Inclusion criteria were as follows: patients who underwent PET/CT examination within 1 week of the initial visit, cancer confirmed by surgery or puncture biopsy pathology, and had complete clinicopathological factors. Patients with metastases in the lung, and those with unavailable clinical pathology and/or imaging data were excluded. The collection of clinicopathological factors included age, sex, family history, smoking history, lymph node metastasis, distant metastasis, TNM staging (I II/III IV), thyroid transcription factor-1 (TTF-1) (− or one + was negative, two or more + was positive), Ki-67 (<25% was low expression, ≥25% was high expression), carcinoembryonic antigen (CEA), tumor diameter, and PET/CT metabolic parameters (Table 1). Telephone follow-up was performed to determine the overall survival of all patients. The follow-up ranged from August 2008 to January 2019. The starting point for overall survival was the date of the PET/CT examination and the end point was defined as the date of telephonic follow-up or death. The differences in largest tumor diameter and metabolic parameters among the different pathological subtypes of invasive lung adenocarcinoma were compared. According to the literature, the different pathological subtypes were divided into two groups: good prognosis group (LPA, PPA, and APA), and poor prognosis group (SPA, MPA). The classification performance of clinicopathological factors and metabolic parameters between good prognosis group and poor prognosis group were analyzed. Survival analysis was performed on patients according to pathological subtype.

**Table 1:** The comparison of clinicopathological factors between good prognosis group and poor prognosis group.

**Characteristics**	**Good prognosis group**	**Poor prognosis group**	***P*-value (FDR-corrected)**
Age, mean ± SD, years	62.17 ± 10.65	61.79 ± 10.45	0.959
Gender, no. (%)			0.411
Male	75 (50.7)	19 (67.9)	
Female	73 (49.3)	9 (32.1)	
Family history, no. (%)			0.959
No	143 (96.6)	27 (96.4)	
Yes	5 (3.4)	1 (0.6)	
Smoking status, no. (%)			0.589
No	104 (70.3)	17 (60.7)	
Yes	44 (29.7)	11 (39.3)	
Distant metastasis, no. (%)			0.585
No	109 (73.6)	24 (85.7)	
Yes	39 (26.4)	4 (14.3)	
Lymph node metastasis, no. (%)			0.959
No	83 (56.5)	15 (53.6)	
Yes	64 (43.5)	13 (46.4)	
Stage, no. (%)			0.625
I/II	91 (61.5)	15 (53.6)	
III/IV	57 (38.5)	13 (46.4)	
TTF-1, no. (%)			0.959
Negative	70 (47.3)	13 (46.4)	
Positive	78 (52.7)	15 (53.6)	
Ki-67, no. (%)			0.394
<25%	83 (89.2)	64 (78.0)	
≥25%	10 (10.8)	18 (22.0)	
CEA, no. (%)			0.917
<5.05	93 (85.3)	55 (82.1)	
≥5.05	16 (14.7)	12 (17.9)	
Diameter, no. (%)			0.479
<3 cm	90 (60.8)	12 (42.9)	
≥3 cm	58 (39.2)	16 (57.1)	

*TTF-1, thyroid transcription factor-1; CEA, carcinoembryonic antigen; FDR, false discovery rate*.

### PET/CT Imaging and Image Analysis

#### Equipment

Patients underwent PET/CT imaging (Biogragh16, Siemens, Erlangen, Germany) using ^18^F-fluorodeoxyglucose (^18^F-FDG) synthesized by the Canadian EBCO TR19 medical cyclotron and chemical synthesis system; radiochemical purity was >95%.

#### Examination Method

The patients fasted 6–8 h before examination. Before examination, height, weight and fasting blood glucose levels were measured, and blood glucose was controlled to below 6.7 mmol/L. Patients were intravenously injected with ^18^F-FDG (5.55 MBq/kg) and quietly rested for 40–60 min, followed by consumption of 500–1,000 ml water, and then emptied their bladder before undergoing whole body PET/CT imaging. The scan ranged from the base of the skull to the upper part of the thigh, data collection included CT and PET scans. The CT scanning parameters were 120 kV (power), 140 mAs (current), and slice thickness and spacing 5 mm. The PET acquisition method was three-dimensional at 3 min/bed. Images were reconstructed using an iterative reconstruction method resulting in CT, PET, and fusion images, which were transferred to a post-processing workstation.

#### Image Processing and Analysis

PET/CT images were analyzed by using visual and semi-quantitative. The lesions on the post-processed images were analyzed by two experienced nuclear medicine attending physicians. Semi-quantitative measurement was performed based on the high FDG metabolic area of the lesion using MS viewer software and by manually delineating region of interest (ROI). ROIs were placed over the primary tumor to measure the maximum standard uptake value (SUVmax) (SUVmax threshold was set to 40%), mean standard uptake value (SUVmean). Calculate the metabolic tumor volume (MTV) (ROI area per layer × layer thickness = volume of each layer, then add the volume of each layer to get MTV). And then calculate tumor-lesion glycolysis (TLG) (TLG = SUVmean × MTV).

### Statistical Analysis

All data were processed using SPSS version 25.0 (IBM Corporation, Armonk, NY, USA). Quantitative data that were normally distributed are expressed as mean ± standard deviation, and the independent sample *t*-test was used for comparison between the two groups. Quantitative data that were not normally distributed are expressed as median (interquartile range), and the Mann-Whitney test was used for comparison between the two groups. Qualitative data are expressed as number and percentage (*n* [%]), and the chi-squared test or Fisher's exact probability method were used for comparison between the two groups. The area under the receiver operating characteristic curve (AUC) for SUVmax, SUVmean, TLG, and MTV index was calculated. Meanwhile, the maximum Youden index was used as the standard to select the optimal cut-off limit value to convert the four quantitative indicators into two-category indicators. Covariates were screened using univariate logistic regression (*P* < 0.20), and further forward likelihood ratio (LR) was used (inclusion test level = 0.05, rejection test level = 0.10) for constructing a multivariate logistic stepwise regression model of predictive factors. Multivariate analysis of predictors was performed to construct the best model, and provide 95% confidence interval (CI) to calculate the AUC, and determine the best cut-off point with the maximum Youden index as the cut-off criterion. The Delong method was used to compare the AUC values of the different models. The false discovery rate (FDR) was used for adjusting the multi comparisons. Survival rates for both groups were analyzed using the Kaplan-Meier method. Survival times are expressed as mean and median and corresponding 95% CI and compared using the log-rank test. A Cox proportional hazard regression model was used to screen covariates, including those with *P* < 0.10 in the univariate analysis, and LR (incorporated with a test level of *P* < 0.05 and a rejection test level of *P* < 0.10). The optimal multivariate Cox regression model was established and the corresponding hazard ratio (HR) and 95% CI were calculated; differences were defined to be statistically significant at *P* < 0.05.

## Results

### Patient Information

**Figure 1:**
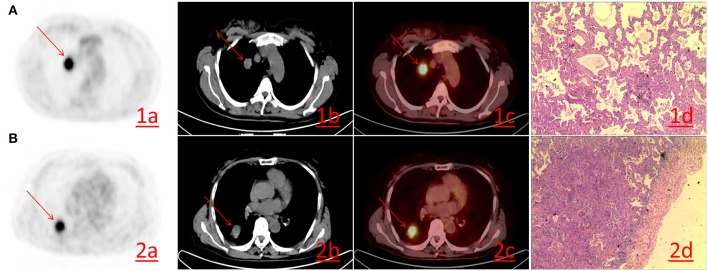
**(A)** A 52-year-old female with lung adenocarcinoma of the right upper lobe. PET/CT revealing lung adenocarcinoma of the right upper lobe, ~19 × 25 mm in size, with increased FDG metabolism. The SUVmax was 10.20 (1a−1c), Hematoxylin-eosin staining (1d) reveals the histological type of acinar predominant adenocarcinoma (HE × 200). **(B)** A 61-year-old female. PET/CT revealing lung adenocarcinoma of the right lower lobe, ~31 × 25 mm in size, FDG metabolism increased and SUVmax was 7.70 (2a−2c). Hematoxylin-eosin staining (2d) reveals the histological type of papillary predominant adenocarcinoma (HE × 200).

Of 176 patients with invasive lung adenocarcinoma included in this study, 94 (53.4%) were male and 82 (46.6%) were female. The mean ages of the patients in good and poor prognosis group were 62.17 ± 10.65 and 61.79 ± 10.45 years, respectively. Other clinicopathological factors and metabolic parameters were shown in Tables 1, 2. The follow-up period was from August 2008 to January 2019. 3 (1.7%) patients were lost to follow-up during the follow-up period, thus leaving 173 patients with complete follow-up data. Among the 173 patients who were followed-up, the median survival time was 47 months [95% CI 44.000–49.000 (range 8–86 months)] and the mean survival time was 47.925 months (95% CI 45.384–50.466). The 1-, 3-, and 5-year overall survival rates were 97.11, 69.36, and 22.54%, respectively. There were 71 (41.04%) patients died during the follow-up period, with median survival time and mean survival time 45.000 months [95% CI 40.00–50.00 (range 8–85 months)] and 45.465 months (95% CI 41.31–49.61), respectively. The 1-, 3-, and 5-year overall survival rates were 92.96, 32.39, and 8.45%, respectively. Representative PET/CT images of two patients with invasive lung adenocarcinoma are shown in Figure 1.

**Table 2:** The comparison of metabolic parameters between good prognosis group and poor prognosis group.

**Metabolic parameters**	**Good prognosis group**	**Poor prognosis group**	***P*-value (FDR-corrected)**
SUVmax, mean ± SD	6.32 ± 4.42	7.97 ± 6.17	0.288
SUVmax, No. (%)			0.016
≥10.17	18 (12.6)	10 (37.0)	
<10.17	125 (87.4)	17 (63.0)	
SUVmean, mean ± SD	3.85 ± 2.75	4.75 ± 3.51	0.300
SUVmean, No. (%)			0.016
≥6.04	16 (11.2)	10 (37.0)	
<6.04	127 (88.8)	17 (63.0)	
TLG, median [P_25_~P_75_] (g)	72.60 ± 161.49	102.85 ± 137.84	0.531
TLG, No. (%), g			0.889
≥5.73	15 (10.5)	2 (7.4)	
<5.73	128 (89.5)	25 (92.6)	
MTVmedian [P_25_~P_75_] (cm^3^)	15.12 ± 26.30	19.01 ± 21.54	0.553
MTV, No. (%) (cm^3^)			0.531
≥4.30	108 (75.5)	23 (85.2)	
<4.30	35 (24.5)	4 (14.8)	

*SUVmax, maximal standard uptake value; SUVmean, mean standard uptake value; MTV, metabolic tumor volume; TLG, total lesion glycolysis; FDR, false discovery rate*.

Differences in the size and metabolic parameters of the lesions were statistically significant among the different pathological subtypes (i.e., Diameter, APA, PPA, LPA, SPA, and MPA) (*P* < 0.05) (Table 3).

**Table 3:** The comparison of size and metabolic parameters of lung adenocarcinoma with different pathological subtypes.

**Subtype**	**Number**	**Diameter (mm)**	**SUVmax**	**SUVmean**	**TLG**	**MTV**
APA	99	26.00 [20.00, 31.50]	5.78 [2.44, 9.08]	2.93 [1.61, 5.44]	6.62 [3.52, 12.87]	6.62 [3.50, 12.87]
PPA	36	27.50 [20.50, 29.25]	6.55 [2.52, 8.21]	2.88 [1.57, 5.04]	25.14 [12.92, 38.39]	8.10 [5.84, 13.18]
LPA	13	21.50 [17.75, 24.50]	2.20 [1.81, 3.16]	1.17 [1.09, 2.88]	10.39 [6.22, 20.49]	8.10 [4.26, 12.68]
SPA	23	27.50 [20.00, 33.50]	8.41 [4.53, 11.44]	5.27 [2.31, 7.01]	52.16 [19.03, 126.15]	10.34 [5.84, 18.97]
MPA	5	27.00 [17.50.83.50]	9.93 [2.74, 16.72]	2.87 [2.83, 5.82]	53.83 [12.85, 213.98]	8.17 [4.51, 50.90]
Total	176	26.00 [20.00, 30.25]	5.99 [2.56, 9.02]	2.90 [1.59, 5.52]	20.91 [11.30, 52.13]	7.46 [3.89, 13.13]
Z		11.408	19.026	19.440	17.696	15.743
*P*-value		0.021	0.001	0.001	0.002	0.008

*APA, acinar predominant adenocarcinoma; PPA, papillary predominant adenocarcinoma; LPA, lepidic predominant adenocarcinoma; SPA, solid predominant adenocarcinoma; MPA, micropapillary predominant adenocarcinoma; SUVmax, maximal standard uptake value; SUVmean, mean standard uptake value; MTV, metabolic tumor volume; TLG, total lesion glycolysis*.

### Prediction of Pathological Subtypes in Good Prognosis Group and Poor Prognosis Group

**Figure 2:**
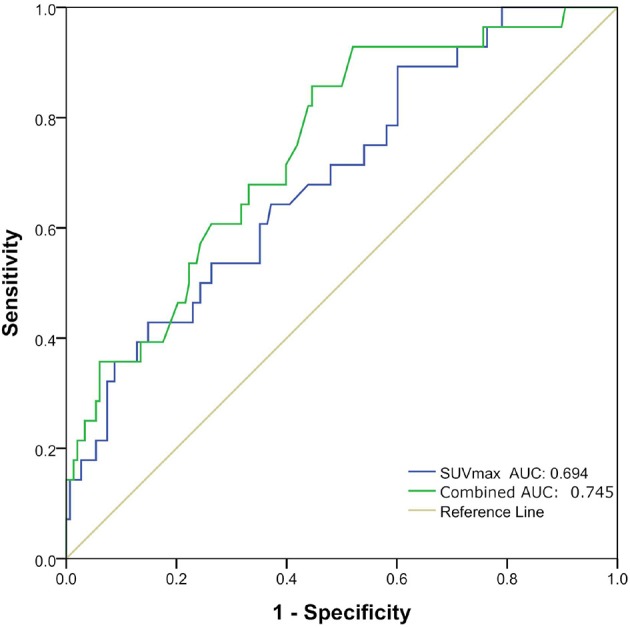
Receiver operating characteristic (ROC) curves of maximal standard uptake value (SUVmax) and combination of three factors (Distant metastasis, Ki-67, and SUVmax) for predicting pathological subtype in good prognosis group and poor prognosis group.

Univariate logistic regression analysis revealed that the Distant metastasis, Ki-67, Diameter and SUVmax, SUVmean were significantly associated with pathological subtypes. The multivariate logistic regression revealed that Distant metastasis, Ki-67, and SUVmax remained independent predictors that predicted pathological subtype. Finally, three predictors (Distant metastasis, Ki-67, and SUVmax) were used to construct a predictive model. The AUC was 0.694 (95%CI0.589–0.798) (*P* = 0.001) when the prediction was performed with the SUVmax, and AUC was 0.745 (95%CI 0.650–0.841) (*P* < 0.001) after combined with clinicopathological factors (Distant metastasis and Ki-67) (Table 4 and Figure 2).

**Table 4:** The univariate and multivariate analysis of various predictive factors for the pathological subtype in invasive lung adenocarcinoma.

**Variables**	**Univariable logistic regression**			**Multivariable logistic regression**		
	**β**	**Odds ratio (95% CI)**	***P***	**β**	**Odds ratio (95% CI)**	***P***
Age	−0.019	0.98 (0.95–1.02)	0.320			
Gender (Male)	0.378	1.41 (0.78–2.57)	0.211			
Family history (Yes)	−10.566	0.000 (0.000–Inf)	0.988			
Smoking status (Yes)	−0.170	1.06 (0.57–1.97)	0.579			
Distant metastasis (Yes)	−0.540	0.58 (0.26–1.29)	0.181	−1.178	0.31 (0.13–0.78)	0.011
Lymph node metastasis (Yes)	0.091	1.15 (0.64–2.05)	0.755			
Stage (III/IV)	0.107	1.12 (0.62–2.01)	0.716			
TTF-1 (Positive)	−0.095	0.94 (0.53–1.69)	0.742			
Ki-67 (≥25%)	0.859	2.54 (1.07–6.04)	0.045	1.012	2.97 (1.16–7.60)	0.031
CEA (≥5.05)	0.238	1.32 (0.57–3.03)	0.570			
Diameter (≥ 3 cm)	0.727	2.07 (0.913–4.70)	0.081			
SUVmax (≥10.17)	1.389	4.08 (1.62–10.29)	0.003	1.733	4.95 (1.75–14.05)	0.001
SUVmean (≥6.04)	1.269	3.62 (1.45–9.01)	0.006			
TLG (≥5.73) (g)	0.383	1.46 (0.32–6.81)	0.624			
MTV (≥4.30) (cm^3^)	0.620	1.86 (0.6–5.86)	0.280			

*SUVmax, maximal standard uptake value; SUVmean, mean standard uptake value; MTV, metabolic tumor volume; TLG, total lesion glycolysis; TTF-1, thyroid transcription factor-1; CEA, carcinoembryonic antigen; CI, confidence interval*.

### Survival Analysis

**Figure 3:**
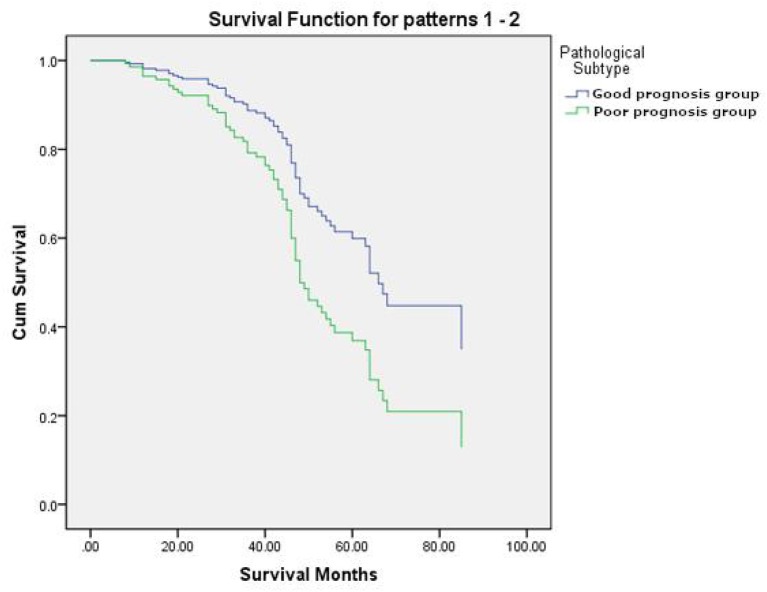
The survival curves of pathological subtypes in good prognosis group and poor prognosis group.

Univariate analysis revealed that Stage (I II versus [vs.] III IV), TTF-1 (negative vs. positive), MTV (<4.30 vs. ≥ 4.30), and pathological subtype (good prognosis group vs. poor prognosis group) were all independent risk factors affecting the overall survival of patients (Table 5). Multivariate analysis revealed that Stage, TTF-1, MTV, and pathological subtype were independent risk factors for patient prognosis. The hazard ratio (HR) of the pathological subtype was 1.948 (95% CI 1.042–3.641), indicating that the risk for death in the pathological subtype of poor prognosis group was 1.948 times that of good prognosis group. The mean survival time of pathological subtypes in good prognosis group was 50.2621 months (95% CI 47.818–52.706), the median survival time was 47.00 months (95% CI 45.000–50.000), the mean survival time of pathological subtypes in poor prognosis group was 35.8214 months (95% CI 27.433–44.159), and the median survival time was 31.50 months (95% CI 23.000–49.000). The prognosis of patients with good prognosis group was better than that of patients with poor prognosis group (Tables 6, 7, and Figure 3).

**Table 5:** The univariate analysis of prognosis in patients with invasive lung adenocarcinoma.

**Prognostic factors**	**HR**	**95%CI**	***P*-value**
Age (<60 vs. ≥60)	1.001	0.978–1.026	0.908
Gender (Males vs. Female)	1.322	0.714–2.446	0.374
Family history (Yes vs. No)	2.383	0.626–9.065	0.203
Smoking status (Yes vs. No)	0.989	0.526–1.858	0.972
Distant metastasis (Yes vs. No)	1.036	0.513–2.091	0.921
Lymph node metastasis (Yes vs. No)	0.765	0.428–1.368	0.367
Stage (I II vs. III IV)	0.156	0.075–0.325	<0.001
TTF-1 (Negative vs. Positive)	1.853	1.026–3.347	0.041
Ki-67 (≥25 vs. <25%)	1.118	0.588–2.126	0.734
CEA (<5.05 vs. ≥5.05)	0.950	0.501–1.802	0.875
Diameter (<3 vs. ≥3 cm)	1.047	0.571–1.921	0.882
SUVmax (<10.17 vs. ≥10.17)	0.442	0.119–1.632	0.220
SUVmean (<6.04 vs. ≥6.04)	3.149	0.884–11.210	0.077
TLG (<5.73 vs. ≥5.73) (g)	0.503	0.171–1.483	0.213
MTV (≥4.30 vs. <4.30) (cm^3^)	1.875	0.979–3.592	0.048
Subtype (Good prognosis group vs. Poor prognosis group)	0.447	0.209–0.955	0.038

*SUVmax, maximal standard uptake value; SUVmean, mean standard uptake value; MTV, metabolic tumor volume; TLG, total lesion glycolysis; TTF-1, thyroid transcription factor-1; CEA, carcinoembryonic antigen; HR, Hazard ratio; CI, confidence interval*.

**Table 6:** The cox proportional hazard regression model for multivariate analysis in patients with invasive lung adenocarcinoma.

**Variables**	**β**	**HR (95% CI)**	***P*-value**
Stage (III/IV)	1.620	5.053 (2.947 ~ 8.663)	<0.001
TTF-1 (Negative)	0.541	1.718 (1.003 ~ 2.941)	0.049
MTV (≥4.30) (cm^3^)	0.569	1.767 (1.012 ~ 3.082)	0.045
Pathological subtype (poor prognosis group)	0.667	1.948 (1.042 ~ 3.641)	0.037

*MTV, metabolic tumor volume; TLG, total lesion glycolysis; TTF-1, thyroid transcription factor-1; CI, confidence interval*.

**Table 7:** The mean and median survival time of pathological subtypes in good prognosis group and poor prognosis group (months).

**Subtypes**	**Mean**	**SE**	**95% CI**	**Median**	**95% CI**
Good prognosis group	50.262	14.89	(47.818–52.706)	47.00	(45.000–50.000)
Poor prognosis group	35.821	21.50	(27.483–44.159)	31.50	(23.000–49.000)

*SE, standard error; CI, confidence interval*.

## Discussion

In our study, tumor size and SUV values were different among the different pathological subtypes of invasive lung adenocarcinoma (i.e., APA, PPA, LPA, SPA, and MPA). This suggested that tumor size and SUV values were valuable for the diagnosis of invasive lung adenocarcinoma of different pathological types. According to the literature, the pathological subtypes of invasive lung adenocarcinoma in our study were divided into good prognosis group and poor prognosis group. And then non-invasive classification for good prognosis group and poor prognosis group were performed using metabolic parameters and clinicopathological factors. The results showed that the predictive model constructed using independent predictors had good classification performance for both groups. The AUC was 0.694 when the prediction was performed with the SUVmax and AUC was 0.745 after combined with clinicopathological factors (Distant metastasis and Ki-67). The current research primarily used the morphological features of nodules, CT quantitative parameters and radiomics to identify pre-invasive and invasive lung adenocarcinoma (25). Li et al. (26) used CT texture features to identify pre-invasive and invasive pulmonary ground-glass nodules, and the AUC was 0.761. Son et al. (27) used quantitative CT parameter analysis of pulmonary ground-glass opacities to distinguish invasive adenocarcinoma from pre-invasive or minimally invasive adenocarcinoma, with an AUC of 0.780. However, we found that using PET/CT metabolic parameters and clinicopathological factors to identify different pathological subtypes of invasive lung adenocarcinoma has been less reported. The predictive model had good classification performance in our study, so, it may be used as a non-invasive method to classify pathological subtypes of different prognosis.

Our study showed that Stage, TTF-1, MTV, and pathological subtypes were independent risk factors for prognosis in patients with invasive lung adenocarcinoma. When invasive lung adenocarcinoma patients are in stage III/IV, TTF-1 expression was negative, MTV ≥ 4.30 and poor prognosis group (SPA, MPA), the patient's risk for death was relatively high. Presently, whether the expression of TTF-1 is related to the prognosis of lung cancer remains controversial. However, some studies (28, 29) suggest that TTF-1 was a very good prognostic indicator, and TTF-1-positive patients have a better prognosis than TTF-1-negative patients. This was consistent with the results of our study. As a parameter that can reflect the metabolic burden of systemic tumors, MTV can stratify patients more effectively, identify high-risk groups, and provide accurate prognosis evaluation compared with other metabolic parameters and related clinical factors. Liao et al. (30) and other studies found that MTV can effectively evaluate the prognosis of stage IV non-small cell lung cancer. Yoo et al. (31) found that MTV and TLG of primary tumors have better value for evaluating the prognosis of patients than other metabolic parameters. In our study, MTV as an independent risk factor was closely related to overall survival and prognosis, and was an important prognostic factor in patients with invasive lung adenocarcinoma. This showed that our study was consistent with previous research. Furthermore, the patients in poor prognosis group (SPA, MPA) had a higher risk for death than those in good prognosis group (LPA, PPA, APA). All of which may provide a little guidance for the prognosis evaluation and treatment strategies for patients.

Our results showed that the prognosis for patients in poor prognosis group was worse than that for those in good prognosis group. Suzuki et al. (32) and other studies reported that 5-year overall survival rates for LPA, APA, PPA, SPA, and MPA of 94, 82, 77, 69, and 57%, respectively, while invasive mucinous adenocarcinomas, and adenocarcinomas with the fetal-type component were 83, and 41%, respectively. The worst prognosis among the five subtypes is MPA, and the worst variant is adenocarcinomas with the fetal-type component. In addition, studies have shown that the prognosis of LPA is the best (33–36), while APA and PPA have a moderate prognosis, and SPA and MPA have the worst prognosis. However, in our study, there were fewer cases of LPA (13 cases) and, as such, LPA was classified into the APA and PPA group. The results of our study revealed that good prognosis group (i.e., LPA, APA, and PPA) had a better prognosis than poor prognosis group (i.e., SPA, MPA), which was consistent with the literature.

Our study possesses some limitations of note, the first of which was its retrospective study and, as such, selection bias was a possibility. Second, the distribution of cases in this study was not balanced, and there were fewer cases in the LPA and MPA groups, therefore, it was not studied according to the three-level classification. Third, our study did not investigate the effects of treatment methods on the prognosis of different pathological subtypes, thus, further study is needed.

In summary, PET/CT metabolic parameters (SUVmax) combined with clinicopathological factors (Distant metastasis and Ki-67) had good classification performance for the different pathological subtypes, which may provide a little guidance for the prognosis evaluation and treatment strategies for patients.

## Data Availability

The raw data supporting the conclusions of this manuscript will be made available by the authors, without undue reservation, to any qualified researcher.

## Ethics Statement

The studies involving human participants were reviewed and approved by The institutional review board of Jinling Hospital, Medical School of Nanjing University approved this retrospective study. Written informed consent for participation was not required for this study in accordance with the national legislation and the institutional requirements.

## Consent for Publication

Patients consented to publishing their images and clinical information.

## Author Contributions

BY conceived the idea of the study and wrote the manuscript. BY, HJ, and SC collected the data. HZ and GL performed image analysis. YG performed the statistical analysis. YG and GL edited and reviewed the manuscript. All the authors discussed the results and commented on the manuscript.

### Conflict of Interest Statement

YG was employed by Siemens Healthineers Ltd. The remaining authors declare that the research was conducted in the absence of any commercial or financial relationships that could be construed as a potential conflict of interest.
